# Density-dependence of nestling immune function and physiological condition in semi-precocial colonial bird: a cross-fostering experiment

**DOI:** 10.1186/s12983-021-00388-y

**Published:** 2021-01-29

**Authors:** Maciej Kamiński, Tomasz Janiszewski, Piotr Indykiewicz, Jacek J. Nowakowski, Jarosław Kowalski, Beata Dulisz, Piotr Minias

**Affiliations:** 1 Department of Biodiversity Studies and Bioeducation, Faculty of Biology and Environmental Protection, University of Lodz, Banacha 1/3, 90-237 Lodz, Poland; 2 Department of Biology and Animal Environment, Faculty of Animal Breeding and Biology, UTP University of Science and Technology, Mazowiecka 28, 85-084 Bydgoszcz, Poland; 3 Department of Ecology and Environmental Protection, Faculty of Biology and Biotechnology, University of Warmia and Mazury in Olsztyn, Plac Lodzki 3, 10-727 Olsztyn, Poland; 4 Gorzewo 7, 09-200, Sierpc, Poland

**Keywords:** Colonial breeding, Immune response, Physiological condition, *Chroicocephalus ridibundus*, Phytohaemagglutinin, PHA, Haemoglobin

## Abstract

**Background:**

Nesting in large aggregations provides several important advantages for colonially breeding birds. However, it also imposes certain costs, associated with facilitated pathogen transmission and social stress. The cost-benefit ratio is not similar for all the birds in a colony and it might be mediated by nest density. To investigate the influence of nest density on cell-mediated immune function and on physiological condition of nestlings, we arranged a cross-fostering experiment in three breeding colonies of black-headed gulls *Chroicocephalus ridibundus*. First, we exchanged eggs between plots of high and low nest density. Afterwards, we performed phytohaemagglutinin (PHA) skin test and we measured blood haemoglobin concentration in nearly 350 nestlings from experimental (exchanged) and control (non-exchanged) groups.

**Results:**

We found that PHA response was lowest in high nest density control group, indicating that depressed immune function of offspring, likely caused by social stress, can be considered as a cost of colonial breeding. Contrastingly, body condition of nestlings was the poorest in low density control group.

**Conclusion:**

Nestlings hatched and raised in high nest density plots did not have higher blood haemoglobin concentration in comparison to other study groups. Furthermore, they were affected with depressed cell mediated immune function, which is possibly driven by combined maternal (corticosteroid hormones deposited in yolk) and environmental (elevated social stress) effects. These results indicate that breeders from high nest densities do not benefit by rising offspring in better quality, in terms of immune function and body condition, although, in the light of previous studies, high nest densities are occupied by birds of higher individual quality, than low density areas. Our study provides a novel insight into the mechanisms of density-dependence that govern fitness of colonially nesting birds.

## Background

Colonial nesting provides birds with significant advantages, for example, substantially diminishing the risk of becoming a target of predator attack, improving the group vigilance, boosting the capability to fend off intruders and spreading the clues of food location [1–4]. These benefits do not come free of charge. The fitness of individuals dwelling in large aggregations of conspecifics is challenged by several factors. The most important ones are intensified intraspecific aggression, social stress and finally — the higher rate of disease transmission [5–7]. Pathogens and macroparasites are an essential threat to the survival and reproductive success [8] and a major constraint to birds sociality [9]. In general, the cost-benefit ratio of reproduction in social groups is not the same for all birds within a colony. High quality individuals can benefit from colonial life, being able to occupy central nesting sites and breed in large densities, where antipredator protection is better. On the other hand, they need to cope with facilitated pathogen transmission and elevated social stress, which can also suppress immune function via corticosteroid hormones [10], but see also [11]. At the same time, individuals of lower quality are often relegated to smaller colonies, peripheral areas and lower nest densities, deprived of basic advantages associated with social breeding, but also freed from its major costs. For these individuals predator threat is bigger, but the social stress and disease transmissions are diminished. This spatial variation in individual quality produces specific spatial variation in fitness-related traits within avian colonies. For example, great cormorants *Phalacrocorax carbo sinensis* breeding in peripheral regions of colony and in lower nest densities raise less male progeny, which are more vulnerable sex [12]. Also, the highest density areas tend to be occupied by older birds in this species [13]. Costs and benefits of social breeding may also depend on the size of reproductive aggregations. In common tern *Sterna hirundo*, pairs nesting in large colony have higher breeding success, but their offspring are in a poorer physiological condition, than in smaller ones [14]. The cross-fostering experiment performed in common tern colonies of different size, but under uniform nesting densities, revealed diminished immune response in tern chicks fostered in a larger colony, demonstrating costs of social stress imposed on immunocompetence [15].

In this study, we aimed to address the influence of breeding pair density on chick immunocompetence (as measured with the phytohaemagglutinin, PHA, skin test) and physiological condition (as measured with blood haemoglobin concentration) in another colonial bird species, black-headed gull *Chroicocephalus ridibundus*. It is a relatively small gull with body mass of 200–300 g, breeding across Palearctic in colonies ranging from several dozens to several thousand breeding pairs [16]. Clutch size is 1–4 eggs, with an average of 2.6–2.8 eggs and the semi-precocial nestlings fledge after approximately 35 days [16]. Sexual size dimorphism is modest, with adult males being ca. 12% heavier than females [17].

Acknowledging our previous results from the common tern study [15], we hypothesized that high nesting densities within a colony may generate similar elevation in social stress as large colonies, resulting in reduced chick induced immune response and physiological condition. On the other hand, since nestlings from high nest densities are exposed on higher pathogen transmission rate, developing enhanced immune response should be adaptive. Maternal antibodies and other substances crucial for immune system are transferred to the offspring [18–20]. But females affected with elevated social stress may deposit in egg yolk stress-associated corticosteroid hormones, which can depress offspring fitness traits [21]. It could induce detectable parental effect on induced immune response of nestlings. To test these hypotheses, we conducted a cross-fostering experiment in three breeding colonies of the black-headed gull, where chicks had their induced immune response and physiological condition measured. In brief, half of the study clutches were exchanged between colony patches of high and low nest densities, resulting in two experimental (foster) and the control (non-exchanged) groups for high and low density. We expected that our experimental design should allow us to separate extrinsic (environmental) effects of nest density on chick immune function and condition from the innate maternal or genetic effects. Assuming that nest density and/or maternal modulation actually influence offspring immunocompetence, we considered three possible scenarios:

1. weaker PHA response of nestlings raised in high densities (independently of their origin) would be consistent with detrimental impact of post-hatching social stress on their immune function;
2. weaker PHA response of nestlings originating from high densities (independently of where they were raised) would be consistent with detrimental maternal effects on their immune function;
3. weaker PHA response of nestlings both originating and raised in high densities (compared not only to nestlings originating and raised in low densities, but also those either originating or raised in high densities) would be consistent with a combined detrimental impact of social stress and maternal effects on their immune function.

## Methods

The cross-fostering experiment was held in 2019, simultaneously in three different (> 80 km apart) breeding colonies of the black-headed gull located in central and northern Poland (Table 1). All colonies were located on islands within lakes. In each colony, after the peak of egg laying period, we fenced several plots with high (1–2 plots per colony, mean area 28.52 m^2^) and low (2–4 plots per colony, mean area 35.8 m^2^) nest density. The mean (harmonic) nest density was 1.06 ± 0.22 (SD) nest/m^2^ and 0.52 ± 0.26 (SD) nest/m^2^ in high and low density plots, respectively, resulting in a two-fold difference. The plots were enclosed with 80 cm high plastic fence with 15 mm mesh. After fencing, a few new nests were built within the plots, but it did not affect the differences in nesting densities between our experimental plots. The plots from each breeding colony were located within similar vegetation patches. Every nest within the plots was recorded and labeled, and each egg was marked with non-toxic permanent marker. A random group of clutches (70–142 clutches per colony; 300 clutches with 618 eggs in total) was exchanged between plots of different density within a colony, in order to form two experimental groups: transferred from high to low nest density (henceforth labelled HL) and transferred from low to high density (LH). We matched pairs of experimental (swapped) clutches according to clutch size and incubation stage (± 2 days), all clutches were swapped as soon as possible after the last egg was laid. We also selected a similar number of unexchanged clutches within plots of each type, which formed the control groups for low (LL) and high density (HH). Mean clutch sizes (2.41–2.70 eggs per nest) and median hatching dates (19–22 May) were similar between all experimental and control groups. The control eggs were handled during plot visits: each egg was picked up, marked and put back to the original nest. The control nests were inspected simultaneously with experimental broods. When the hatching commenced, the plots were visited regularly to ring the hatchlings and assign them to their natal nests before they became mobile. Within the fenced plots the nestlings were able to wander freely and interact with other individuals. Age of all hatchings subjected to the experiment was determined with an accuracy of ±2 days. Any chicks that could not be assigned to their natal nests with certainty were excluded from further treatment. Due to relatively high egg losses and chick mortality in some of the study plots and inability to assign all chicks to their natal nests with certainty, the number of chicks subjected to experimental procedures were much lower (38.7%) than the original number of exchanged and control eggs.

**Table 1:** Information on black-headed gull colonies in which the study was conducted

Colony	Location	No. breeding pairs	No. exchanged clutches	High density area		Low density area	
				No. plots	Exp./control chicks	No. plots	Exp./control chicks
Rynskie	53°55′11″N 21°30′48″E	5000	88	2	6/6	2	34/21
Przykona	51°58′26″N 18°36′51″E	3000	70	1	31/33	2	17/13
Skoki	52°36′23″N 19°23′07″E	4100	142	2	59/38	4	35/56

PHA skin test was used to assess induced immune response of nestlings. The PHA, due to mitogenic properties, stimulates T-lymphocytes to accumulate and aggregate in the spot of injection. This visibly manifests in swelling of injected spot and daily thickness increase was traditionally interpreted as a proxy of T-cell mediated immune response, although there is a growing body of evidence showing that the leukocyte reaction upon PHA is much more complex and also involves other types of immune cells and subsequent inflammation [22–24]. Nevertheless, due to its simplicity, PHA skin test has been extensively used in ecological studies on wild animals to assess immunocompetence [25–27]. For injections we used 0.2 mg PHA (Phytohaemagglutinin PHA-P L8754, Sigma-Aldrich Co., St. Louis, MO, USA) dissolved in 0.05 ml of phosphate-buffered saline (PBS; Sigma-Aldrich Co.). After removing the down feathers, the solution was injected in left wing patagium, using syringe with 0.33 mm gauge needle. The thickness of patagium was measured (to the nearest 0.01 mm) with pressure-sensitive micrometer (Mitutoyo, Kawasaki, Japan) before the injection and after 24 h. The measurements were repeated three times and averaged. The mean difference of thickness before and after PHA injection was used as an index of immune response. In one case, the nestling did not developed any swelling response to PHA (probably due to technical problems with injection) and was excluded from the analyses. Eventually, after accounting for nest failures and chicks mortality, we performed PHA skin test on 349 nestlings in three colonies jointly (182 fostered individuals and 167 in control groups from 115 and 103 broods respectively; 67–188 nestlings per colony). The mean age of nestlings was 15.39 ± 4.39 (SD) days.

To assess physiological condition of nestlings, we measured whole-blood haemoglobin concentration. Blood haemoglobin concentration is related with the ability to satisfy oxygen requirements, hence it is a robust indicator of physiological condition and individual quality (reviewed in [28]). Just before conducting the PHA skin test, we collected a small droplet of blood into special microcuvettes by puncturing right wing ulnar vein with disposable needle. The blood haemoglobin concentration was instantly analyzed in a portable photometer (HemoCue Hb 201+, HemoCue, Sweden). For technical reasons, we failed to measure blood haemoglobin concentration in 7 birds subjected to PHA skin test (*N* = 342).

All computations were performed in software environment R v. 3.6.2 [29]. We used general linear mixed models, run with *lmer* function from the *lme4* package [30], to test for the influence of nesting density on PHA response and blood haemoglobin concentration (Table 2). The fixed factor of nesting density had four levels, including control and experimental (cross-foster) nestlings from low and high densities. The *lmerTest* package [31] was used to infer statistical significance (*P* values) and comparisons for all study groups. Since both immune function and blood oxygen-carrying capacity are expected to gradually develop during the nestling period [32, 33], we included chick age as a covariate and, additionally, we tested for its interaction with nest density. To test for a possible non-linearity of age effects we also added the squared term of age to each model (removed if non-significant). In both models we entered nest identity as a random factor. Due to highly unbalanced sample sizes among experimental groups from particular breeding colonies (Table 1), we did not test for an interaction between nest density and colony. Instead, we used colony identity also as a random factor. The PHA response was log-transformed due to high right-skewness (1.04). The models were fitted with restricted maximum likelihood. With *MuMIn* package [34], we inferred marginal and conditional R^2^ (representing variance explained by fixed effects only and total variance explained by fixed and random effects, respectively) for all the models (reported in table captions; Tables 2 and 3). All figures were prepared with *ggplot2* package [35].

**Table 2:** The effect of study group (LL – low nest density control group, LH – low-high nest density fostered group, HL – high-low nest density fostered group, HH – high nest density control group) and nestling age on PHA response, as assessed with general linear mixed model (restricted maximum likelihood). The breeding colony and nest identities were used as random factors. Significant variables are shown in bold. The marginal and conditional R^2^ for the model are 0.041 and 0.322 respectively

Explanatory variable	β coefficient ± standard error	df	t	*P*
**Intercept**	**0.64 ± 0.07**	**12.1**	**9.93**	**< 0.0001**
Study group (HL vs. HH)	0.07 ± 0.04	216.6	1.95	0.052
**Study group (LH vs. HH)**	**0.09 ± 0.04**	**197.8**	**2.66**	**0.01**
**Study group (LL vs. HH)**	**0.07 ± 0.04**	**202.1**	**2.06**	**0.04**
Study group (HL vs. LH)	−0.02 ± 0.04	203.0	−0.62	0.54
Study group (HL vs. LL)	−0.002 ± 0.04	207.9	− 0.07	0.99
Study group (LH vs. LL)	0.02 ± 0.03	191.0	0.56	0.58
**Age**	**−0.01 ± 0.003**	**297.8**	**−2.13**	**0.03**

**Table 3:** The effect of study group (LL – low nest density control group, LH – low-high nest density fostered group, HL – high-low nest density fostered group, HH – high nest density control group) and nestling age on blood haemoglobin concentration, as assessed with general linear mixed model fitted (maximum likelihood). The breeding colony and nest identities were used as random factors. Significant variables are shown in bold. The marginal and conditional R^2^ for the model are 0.062 and 0.520 respectively

Explanatory variable	β coefficient ± standard error	df	t	*P*
**Intercept**	**81.26 ± 10.22**	**44.56**	**7.95**	**< 0.0001**
Study group (LH vs. LL)	5.00 ± 2.63	163.26	1.90	0.06
**Study group (HL vs. LL)**	**7.05 ± 2.61**	**175.58**	**2.71**	**0.01**
Study group (HH vs. LL)	2.20 ± 2.73	170.74	0.80	0.42
Study group (HL vs. LH)	2.05 ± 2.68	175.2	0.77	0.44
Study group (HH vs. HL)	−4.86 ± 2.77	188.5	−1.75	0.08
Study group (HH vs. LH)	−2.81 ± 2.71	172.6	− 1.04	0.30
**Age**	**3.52 ± 1.11**	**333.46**	**3.18**	**0.002**
**Squared age**	**−0.09 ± 0.03**	**323.87**	**−2.65**	**0.009**

## Results

**Fig. 1:**
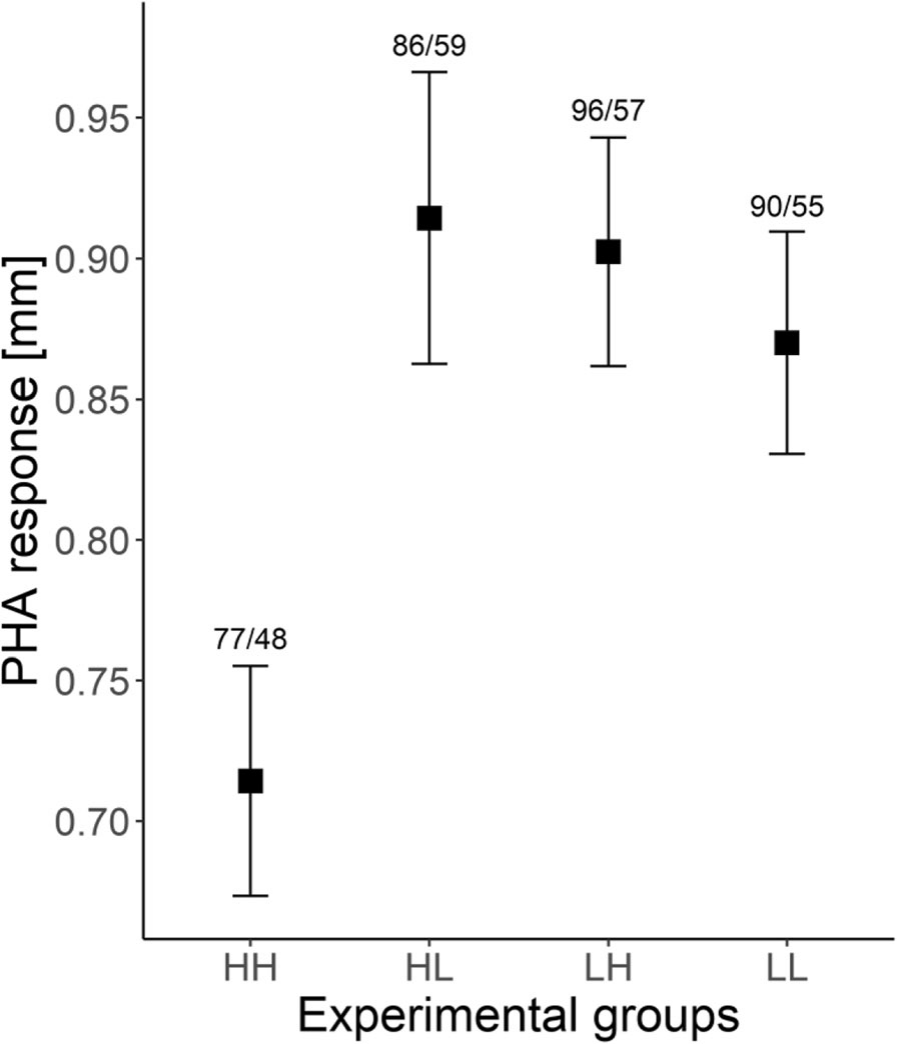
Comparison of PHA response in experimental groups (LL – low nest density control group, LH – low-high nest density fostered group, HL – high-low nest density fostered group, HH – high nest density control group) of black-headed gulls nestlings. Black squares indicate mean value and whiskers represent standard error. The numbers above whiskers represent number of nestlings/number of broods

Our results showed that nestlings from high density control group (HH) had significantly lower PHA response (Fig. 1, Table 2) than birds from LL and LH groups (both *P* < 0.05, Table 2) and nearly significantly lower than birds from HL group, (*P* = 0.052, Table 2). The age was also identified as a significant predictor of PHA response, as younger birds where more likely to develop thicker response in injection spot (β = − 0.006 ± 0.003, *P* = 0.034). However, the model with an interaction between age and nest density showed that the relationship of PHA response with age was significant only in the LH group (β = − 0.016 ± 0.005, *P* = 0.0006), remaining non-significant in the others (*P* > 0.05). The quadratic effect of age was not significant and was removed from the final model.

**Fig. 2:**
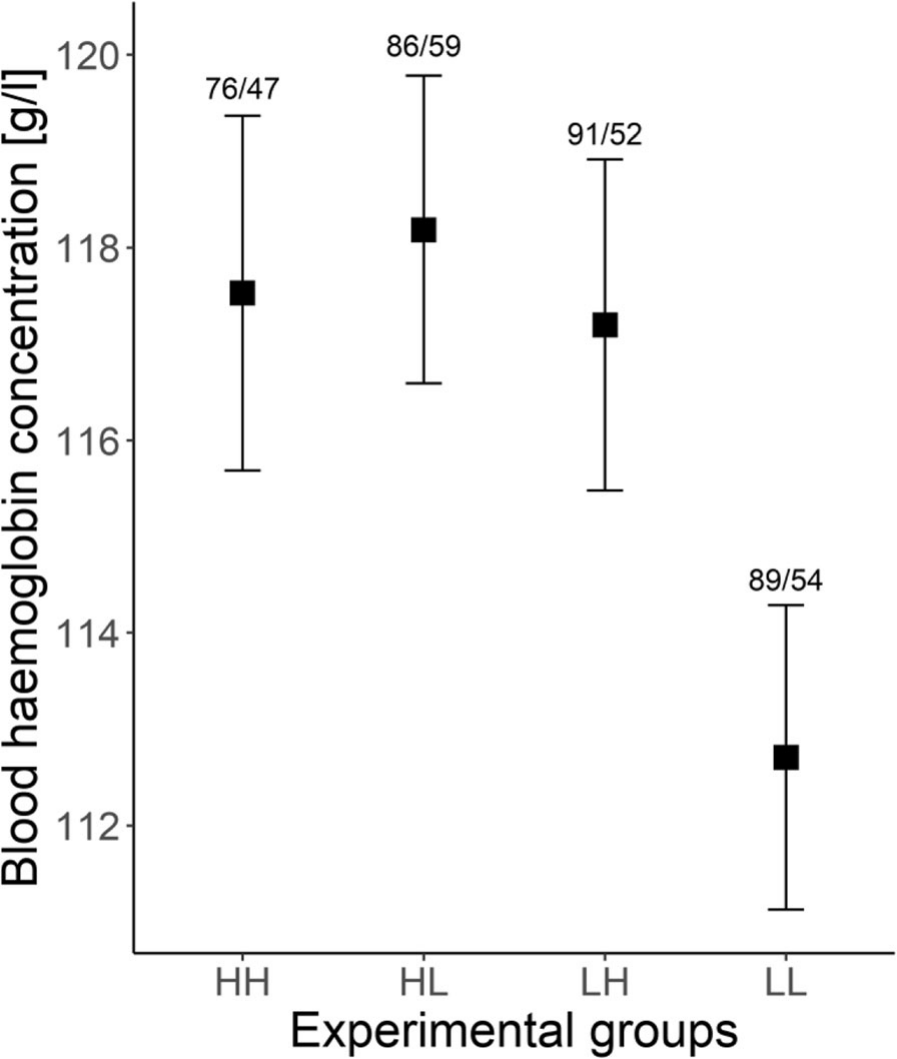
Comparison of blood haemoglobin concentration in experimental groups (LL – low nest density control group, LH – low-high nest density fostered group, HL – high-low nest density fostered group, HH – high nest density control group) of black-headed gulls nestlings. Black squares indicate mean value and whiskers represent standard error. The numbers above whiskers represent number of nestlings/number of broods

In contrast to PHA response, blood haemoglobin concentration was lowest in the low density control group (Fig. 2) and it significantly differed from the HL experimental group (β = 7.052 ± 2.607, *P* = 0.008), while the difference with LH group was close to the significance threshold (β = 5.000 ± 2.626, *P* = 0.059). The difference between the two (high density and low density) control groups was not significant. Nestling age was positively associated with blood haemoglobin concentration, but relationship was non-linear, as indicated by significant quadratic effect, which was retained in the final model (Table 3). The interaction between age and nest density was non-significant (*P* = 0.695).

## Discussion

Our study provided experimental evidence for density-dependence in the immune response of black-headed gull nestlings. The results revealed that nestlings hatched from eggs originally laid in high densities and subsequently raised in high density areas of gull colonies (HH group) developed less intense response to PHA injection (Fig. 1). The difference between HH and HL was marginally non-significant, but we deem it biologically meaningful. The immune response pattern was strikingly contrasting with variation in blood haemoglobin concentrations of nestlings, which were lowest in the low density control group (Fig. 2). Because the results for both PHA challenge and haemoglobin measurements were not congruous across pre-natal or rearing nest density, but emerged in control groups (HH for immune response and LL for haemoglobin concentration), we presume that this is the result of combined maternal and environmental influence.

The major finding from our study seems to be consistent with the results from a previous cross-fostering experiment conducted in common tern colonies [15], where nestlings raised in larger reproductive groups (but under similar nesting densities) showed depressed PHA response. Similar, although non-experimental, variation in the immune response between different colony sizes was reported for the nestlings of Magellanic penguin *Spheniscus magellanicus* [10] and attributed to the negative effect of social stress. The elevated social stress is recognized as a possible cost of avian coloniality [14]. Consistently with this hypothesis, it has been shown in black-headed gull that birds nesting in central colony area (areas of higher nest density) are affected by stronger social stress, as indicated by elevated heterophile/lymphocyte (H/L) ratios [36]. In general, changes in H/L ratios are induced by exposure to external stressor (e.g. the risk of injury) and are mediated by the corticosteroid hormones [37]. The corticosteroids inhibit inflammatory response and deplete T-cells in peripheral blood [38] and, hence, prolonged exposure to stress may suppress immune functions. The short-term stressful events are unlikely to affect induced immune response and persistent exposure to stressors is often required to affect immunity [39]. In this study, reduced immune reaction occurred in gull nestlings that originated and were raised in high nest density (HH). Remarkably, nestlings from a cross-fostered experimental group, which originated from low, but were raised in high nest density (LH) had significantly higher PHA response, comparing to high density control group. Thus, exposure to social stress during the nestling stage is likely insufficient to explain low immune response exclusively in the HH group and our results allow to rule out predicted scenarios 1 and 2 (see Background). Taking this into account, we speculate that the suppression of immune function, which we observed in high density control group, could be attributed to a combined effect of two mechanisms that may induce elevated social stress (scenario 3): direct influence on nestlings dwelling in high nest density (via higher rate of antagonistic interactions with other chicks and non-parental adults) and indirect influence via increased levels of maternal corticosteroids deposited in eggs laid by females exposed to high social stress. In fact, it is known that maternal corticosteroid hormones are transferred to egg yolk [40] and they can detrimentally affect PHA response in nestlings [41]. Unfortunately, we do not have any available data on the density-related variation in corticosteroid hormones of nesting females and in eggs they laid. According to the environmental matching hypothesis [42, 43], the offspring should have increased fitness when facing environmental conditions matching those, experienced by their mothers. If so, the potential adverse influence of high nest density should be mitigated. Since in the HH group the nestlings were matching the maternal environment and the mismatched experimental groups were not affected by depressed immune response, we do not find evidence supporting this hypothesis, at least, in short-term perspective during pre-fledging life period. Also, albeit we controlled for the colony effect in our analyses, we acknowledge that several local ecological factors, like food availability or overall colony size (our study colonies were relatively large), might shape the trade-off balance and also influence investments in immune functions in different breeding colonies. Black-headed gull is a dimorphic species and induced immune response may show sex-specific patterns [44, 45]. In birds, males - when being the larger sex, as it is in black-headed gull - often tend to be more vulnerable to adverse conditions [12, 46]. Nonetheless, we presume that the results of our experiment should not derive from sex-specific variation, since only limited difference was reported in PHA response between male and female black-headed gulls [47] and nestling sex ratio is unlikely to deviate from parity in this species [48].

The nestlings from low density control group (LL) were in the poorest physiological condition, as indicated by the blood haemoglobin concentration (Fig. 2). However, the only significant difference in the model was found between LL and HL (eggs laid in high densities, nestlings raised in low densities) experimental group. This may indicate genetic influence or maternal effect on blood haemoglobin level. In general, birds nesting in more attractive, high-density areas are expected to be of higher phenotypic and genetic quality [13, 49], which can have a positive effect on the quality of offspring. In fact, central areas of black headed gull colonies were previously shown to be occupied by adult birds with higher blood haemoglobin concentration and higher size-corrected body mass, indicating their better individual quality in terms of body condition [36]. The artificial reduction of colony size in herring gull *Larus argentatus* resulted in improvement of body condition of the breeders [50], which shows constraint inflicted by competition with conspecifics. The parental status should influence their offspring condition as well. We are not aware of any study addressing heritability of blood haemoglobin concentration in birds, but other haematic indicators of condition in wild birds show some limited, but significant heritability, although they also tend to strongly depend on environmental factors [51, 52]. Study of another colonial and semi-precocial larid, the common tern, demonstrated that progeny from large colony had higher survival rate, but was in generally poorer physiological condition, with lower blood haemoglobin concentration, slower growth rate and elevated social stress (elevated H/L ratios) [14]. Nevertheless, contrasting patterns of density-dependence in the immune function and haemoglobin concentration suggest that some fitness-related traits of nestlings can be more vulnerable to social stress (PHA response) than the others (haemoglobin concentration), implying that opposing selective pressures may be associated with different nesting densities in colonial birds. At the same time, one should bear in mind that, despite having a negative effect on immune function, high density environment may have positive impact on other fitness-related traits, which were not examined in this study. For example, herring gull *Larus argentatus* chicks from dense colony regions had higher survival rate and this advantage was especially apparent in the last-hatched nestlings [53]. It was also reported that nestlings from high nest density areas had faster growth rate, but the scope of the study covered only first 9 days of life [53].

## Conclusions

In conclusion, the results of our study indicate that breeders nesting in more favorable (in terms of antipredator protection) high-density colony areas do not visibly benefit in raising offspring of better physiological condition, as well as of better immunological state. Quite the opposite, it appears that depressed cell-mediated immune function of nestlings should be regarded as a cost of hatching and being raised in high density environment, possibly traded for other benefits deserving closer scrutiny – like higher survival due to better protection from predation. Moreover, we found evidence that negative effects of colonial nesting imposed on immune response arise from a combined impact of post-hatching social environment and pre-laying maternal effects. The question of fitness trade-offs in colonial breeders, especially with reference to immunocompetence, still merits further investigation.

## Data Availability

The data set used in the study is available from the corresponding author upon reasonable request.
